# A Graph Theoretical Approach to Study the Organization of the Cortical Networks during Different Mathematical Tasks

**DOI:** 10.1371/journal.pone.0071800

**Published:** 2013-08-19

**Authors:** Manousos A. Klados, Kassia Kanatsouli, Ioannis Antoniou, Fabio Babiloni, Vassiliki Tsirka, Panagiotis D. Bamidis, Sifis Micheloyannis

**Affiliations:** 1 Lab of Medical Informatics, Medical School, Aristotle University of Thessaloniki, Thessaloniki, Greece; 2 Neurophysioloical Research Laboratory (L. Widén), Medical School, University of Crete, Heraklion, Greece; 3 Department of Mathematics, Aristotle University of Thessaloniki, Thessaloniki, Greece; 4 Department of Physiology and Pharmacology, University of Rome “Sapienza,” Rome, Italy; University of Zaragoza, Spain

## Abstract

The two core systems of mathematical processing (subitizing and retrieval) as well as their functionality are already known and published. In this study we have used graph theory to compare the brain network organization of these two core systems in the cortical layer during difficult calculations. We have examined separately all the EEG frequency bands in healthy young individuals and we found that the network organization at rest, as well as during mathematical tasks has the characteristics of Small World Networks for all the bands, which is the optimum organization required for efficient information processing. The different mathematical stimuli provoked changes in the graph parameters of different frequency bands, especially the low frequency bands. More specific, in Delta band the induced network increases it’s local and global efficiency during the transition from subitizing to retrieval system, while results suggest that difficult mathematics provoke networks with higher cliquish organization due to more specific demands. The network of the Theta band follows the same pattern as before, having high nodal and remote organization during difficult mathematics. Also the spatial distribution of the network’s weights revealed more prominent connections in frontoparietal regions, revealing the working memory load due to the engagement of the retrieval system. The cortical networks of the alpha brainwaves were also more efficient, both locally and globally, during difficult mathematics, while the fact that alpha’s network was more dense on the frontparietal regions as well, reveals the engagement of the retrieval system again. Concluding, this study gives more evidences regarding the interaction of the two core systems, exploiting the produced functional networks of the cerebral cortex, especially for the difficult mathematics.

## Introduction

The ability to process numbers and perform more complex arithmetic tasks is generally believed to be supported by at least three component systems, each primarily responsible for estimating numerical quantities, remembering and retrieving math facts, and learning and applying calculation strategies, respectively.

Numerical estimation (also known as subitizing) incorporates the ability to appreciate quantities and establish a sense of numerocity. Accurate number estimation (without the serial process of actual counting) is typically observed with up to 4–5 items [1] and is also found in non-human animals [2], [3]. Moreover, infants by the first year of their life have the subitizing ability, while the cortical structures located in the posterior parietal and parieto-occipital region, bilaterally, appear to be the critical components of this system [3]–[6].

A key component of the second system, responsible for learning and retrieving math facts, appears to be located in the left angular gyrus (lAG) [7]–[9]. According to one view [10] the lAG is involved in the retrieval of verbally stored arithmetic facts (such as multiplication facts or simple additions) by operating within the language system through its well-established connections to the left temporo-parietal cortex [11]. This region is more active in exact than in approximate calculation. During exact calculation the lAG shows higher activation for arithmetical operations related to rote verbal memory arithmetic facts. Thus, lAG shows more activation for multiplication tables in relation to subtraction or number comparison [10], [12]. Jost et al. in a functional Magnetic Resonance Imaging (fMRI) and electroencephalographic (EEG) study found that that lAG is engaged in arithmetic fact retrieval together with frontal structures [13]. Small sized problems (with a sum below 10) activate the lAG more than the large ones and this is explained as the result that these operations are stored in rote verbal memory like the multiplication tables [10], [12]. A comparison of the performance of a large group of left hemispheric patients, on language comprehension and arithmetic, showed a relationship between language and arithmetic. In this study, behavioral and anatomical data were indicative of partially overlapping networks (including lAG) sub-serving language and arithmetic [14]. Individuals with higher mathematical competence show higher activation of the lAG while solving multiplication problems as a result of reliance of language mediated processes and arithmetic problem solving [12].

In contrast to the other findings, reduced activation of the lAG has been reported in perfect (and faster) compared to imperfect performers during arithmetical processing, and in easier problems rather than the more difficult ones [12], [15]–[17]. This is difficult to explain since most studies show higher activation of lAG during arithmetic. One explanation was that this deactivation is related to the default mode network deactivation since lAG belongs to the default mode network [12], but this explanation does not cover all the findings. Grabner used fMRI and the associate confusion effect which allows the dissociate effects of task difficulty and task-related arithmetic processes on lAG activation [18]. He ruled out that lAG activations are an epiphenomenon of task difficulty which affects the default mode network and concluded that lAG supports automatic mappings between mathematical symbols and solutions stored in long term memory.

Mathematics and language seems to have an overlap in their brain regions supporting the performance for both functionalities [14], [19], [20]. In the triple-code theory, [21] it is proposed that there are three regions for numerical quantities: the left perisylvian language network, a ventral occipito-parietal region for visual representation of digits, and the horizontal Intraparietal sulcus (IPS) for processing of visual representation of digits [21]. Except the studies focusing mainly on the frontal and parietal lobes and their role in mathematics, several other studies exist for differences of the basic arithmetic operation, detailed function of IPS etc. These provide interesting insights but they are far from explaining the brain function during higher mathematical processing.

A question that arises, concerns the identification of the mechanism and how it supports “real mathematics” like fractions, negative numbers, negative roots, calculus etc, while another one concerns the series of events in the brain during advanced mathematical processing. Both questions are very difficult to answer; nevertheless the IPS has been recognized as a hub region for mathematics. Additionally parietal-frontal networks as well as some basal ganglia and networks associated with long-term and working memory, are engaged in mathematics and especially in very difficult problem solving and only in expert calculators [7]. The IPS is activated when numbers are presented in various notations or spoken number words and it is claimed to contain a specialized subsystem for numbers [20].

Probably, what it is lacking from the previous analyses is a proper consideration of the fact that often cortical regions become activated in concert (i.e. in a network fashion) even during simple tasks. This could explain why a particular cortical region appears predominantly in one particular task, while almost disappearing in another apparently similar one. In recent years, the study of how the different cortical regions could be functionally connected during a specific task has been investigated with several neuroimaging technologies [13], [22]–[24]. Between the different neuroimaging methodologies, neuroelectrical imaging [25] is one of the most suited for the estimation of the activation of the cerebral cortical networks during a task performed while recording the Electroencephalogram (EEG).

There have been several attempts to establish associations between various cognitive processes and electrophysiological changes in specific EEG frequency bands. The majority of the studies have focused on the alpha band demonstrating reduction in alpha power with task performance [25]. Moreover, electrical events occurring in the delta band may be involved in the large-scale cortical integration, whereas increases in theta power have been linked with memory encoding and retrieval. Brain activity in the beta band is often regionally constrained and may play a role in the direction and maintenance of attention, especially during motor tasks. Finally, synchronization in the gamma band has been put forward as a correlate of information binding across nearby cortical sites. With respect to mathematical thinking, the majority of the electrophysiological studies, employ Event-Related Potentials (ERP) and to a lesser degree Event-Related Desynchronization/Synchronization (ERD/S).

In addition to ERPs and ERD/S, EEG data may shed light into the temporal dynamics of brain networks that become active during a task performance. In a previous study employing relatively simple calculation tasks, we reported band- and region-specific EEG power spectrum changes during performance of mental multiplication tasks as compared to control tasks (dot or number viewing) [26]. Specifically, we found increases in the gamma power over the left hemisphere, reduced alpha2 power at central and parietal areas, and increased theta power at frontal locations. These results were consistent with evidence obtained from other imaging modalities of increased levels of neuronal signaling within a distributed set of neuronal networks, primarily in left frontal and parietal areas, but also in the right fronto-parietal area associated with retrieval of simple mathematical facts (e.g. multiplication tables), which are typically stored in the form of verbal representations. Despite the significant difference in difficulty between the two calculation tasks (corroborated by performance data), corresponding differences in regional spectral power have been scarce.

To the best of our knowledge, this is the first time that these findings were extended in the time and frequency domain by modeling the associations between the cortical dipoles obtained, using the inverse solutions, by the signals recorded at different electrode sites. Network theory (graph theory) was employed in order to derive patterns of network organization which may in turn reflect underlying functional brain networks [27]. Our approach capitalizes the recent application of graph theory which estimates the regional activity of the cerebral cortex obtained by the EEG data [28]. As it was mentioned before, this approach was adopted in order to investigate the changes of the cortical networks’ organization as a function of (a) task, contrasting two multiplication tasks (one and two digit multiplications) with two control tasks (visual fixation and a task involving countable random dot arrays), and (b) calculation difficulty. The latter goal was pursued by contrasting the two multiplication tasks which differed on difficulty and complexity level. Whereas performance of the single digit multiplication task may rely on math fact retrieval, the harder, two-digit multiplication task requires implementation and specific calculation strategies in addition to retrieval of simple math facts (times tables).

In this piece of work weighted graphs have been used in order to provide a more realistic representation of the cortical networks, whereby a “weight” is computed for as an index of the strength of each connection [29]. Well-established parameters were further computed describing local and widespread organization of the networks, including Global Efficiency (Eglob), Local Efficiency (Eloc), Node Strength (NS), and the “Small World Network” (SWN) index [30]. It is worth noting that high SWN values characterize networks displaying a high clustering coefficient and short path length, which all are properties of real networks that show optimum organization.

In particular, this study investigates the functional networks subserving the two core systems of the mathematical cognition, as well as their modulation according to the varying difficulty of a certain mathematical task. Moreover this study comes to shed light on the role of each particular EEG frequency band and it’s ability to describe the difficulty of the proposed mathematical stimuli.

## Materials and Methods

### Participants

Twenty four right handed medical students of the University of Crete were recruited for this study (12 and 12 females with a mean age of 23 years (SD = 2.1 years). Participants with a history of psychiatric or neurological illness or under medication were excluded from the study. All participants had normal (10/10) or corrected to normal vision. Participants were asked to avoid alcohol intake on the day before and caffeine consumption on the day of the experiment; they were also asked to sleep as adequately and comfortably as possible on the night before. Recordings were performed during the morning. All participants signed an informed consent form, while the experimental protocol was approved by the ethics committee of the Department of Medicine of the University of Crete.

### EEG Recordings

EEG recordings were performed in a dark, electrically shielded and sound attenuated chamber. Participants were lying in a comfortable chair and the stimuli were presented on a laptop screen located about 80 cm in front of the individual. EEG signals were recorded with 32 electrodes placed on the scalp according to the 10/10 international system (FP2, F4, FC4, C4, CP4, P4, O2, F8, FT8, Fz, FCz, Cz, CPz, Pz, Oz, FP1, F3, FC3, C3, CP3, C3, F3, FC3, C3, CP3, P3, O1, F7, FT7, and PO7, referenced to linked earlobes). From these 32 electrodes, one (1) was used as the trigger (CPz), one (1) for the grounding (AFz), and two (2) for the detection of eye-movements (VEOG) thereby resulting in 28 electrodes used for the EEG recordings. The signals were amplified, digitized at 500 Hz and online filtered between 1 and 200 Hz.

### Stimuli and Tasks

**Figure 1:**
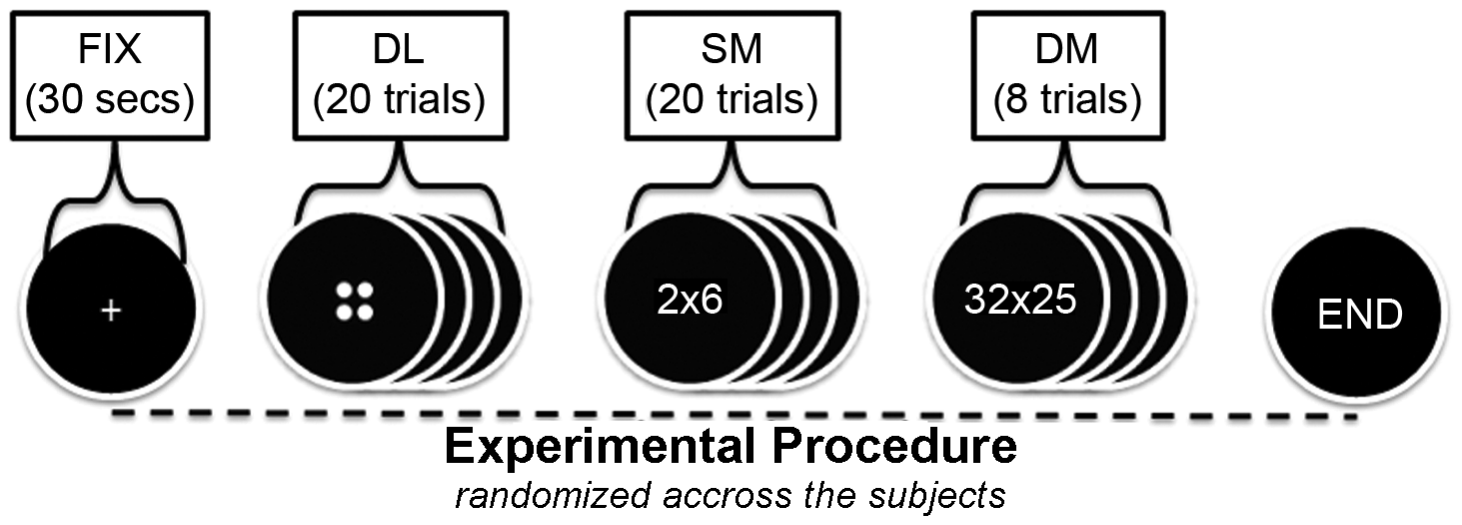
Experimental Design: In this figure the experimental flow is being observed. All the subjects took place in the current study, were exposed to these four different tasks namely FIX, DL, SM, and DM. The first task was pseudoradomly chosen for each individual.

EEG data were acquired during performance of four tasks (Fixation, Dot looking, Simple Multiplication, and Difficult Multiplication). During Fixation (FIX) a small white cross appeared at the center of a dark screen for 30 s (control condition). During the dot looking (DL) block participants passively viewed 2–4 dots for 20 trials lasting 1 second each. The Simple Multiplication (SM) task included 20 trials of single-digit multiplications (e.g. 3×3), while the DM task included 8 trials of two-digit difficult multiplications (e.g. 35×72) (see Figure 1). For the simple multiplication tables we asked the individuals (students of medicine) if they had any difficulty or question. Responses were provided orally after the DM task (for analysis we chose pieces of 10 s duration, without visible artifacts and with correct answers and all the signals were further cleared by ocular artifacts using the REGICA [31] plugin for the EEGLAB toolbox [32]). No response was required during dot looking and fixation, while for the SM and DM tasks, students were not restricted to reply within a prescribed time period.

### Cortical Activity

**Figure 2:**
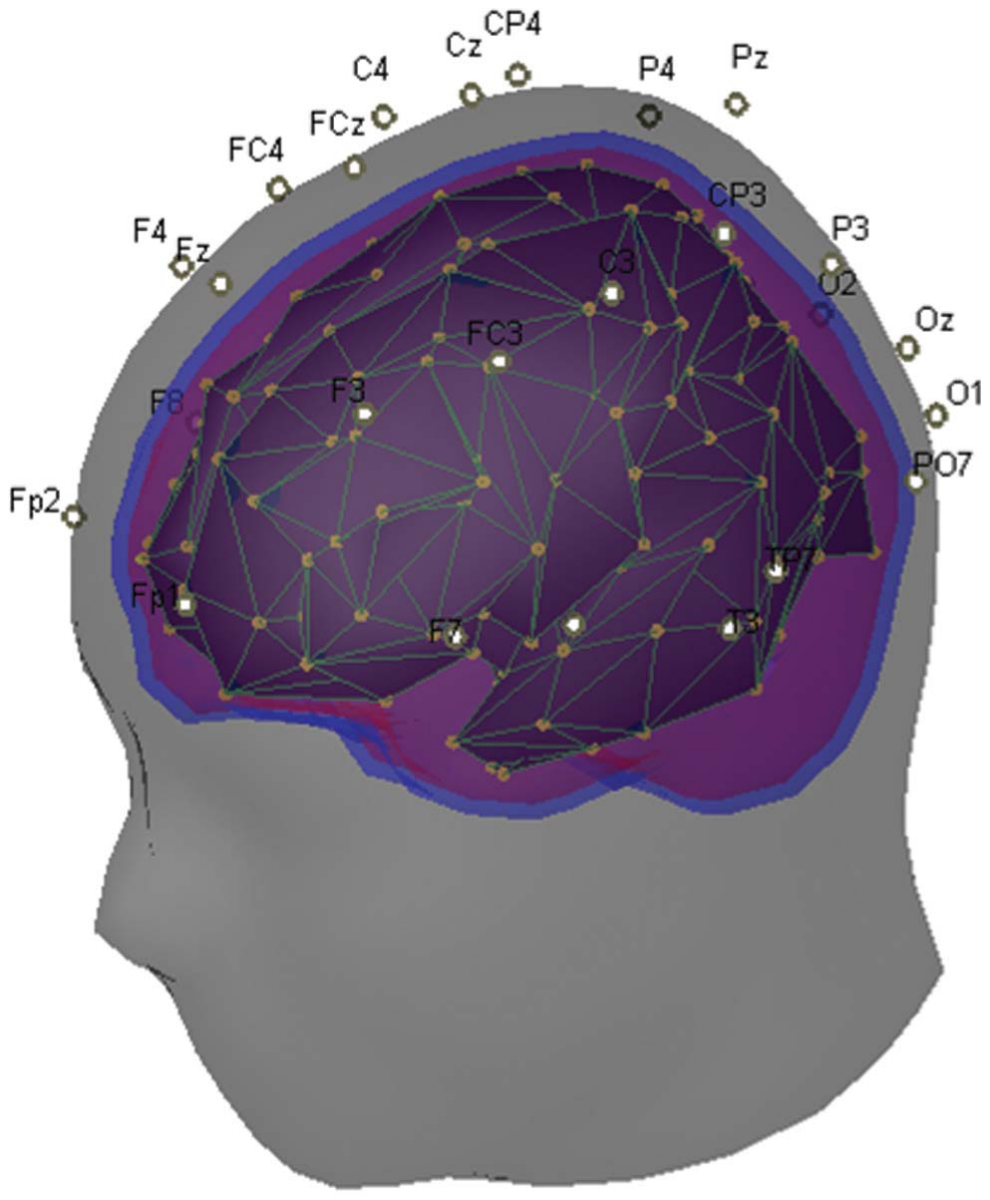
Head Model: According to our head model, the electrodes’ positions and the cortical dipoles (yellow dots) are known. Since the conductivities of the head (gray layer), the outer skull (blue layer) and the inner skull (purple layer) are also known, we can compute the mapping from out 28 electrodes to 258 cortical dipoles.

EEG records the activity of the cortical dipoles oriented in tangential or radial directions regarding to the scalp surface. Despite that the variation of the electrical conductivity among the different head compartments leads to the volume conduction problem, which is very serious drawback of the functional connectivity analysis [33]. To face this problem, the cortical activity was estimated from the 28 EEG signals, by adopting a realistic head model, which describes the different electrical conductivities of the head structures and the sensors’ geometry, a cortical dipole model, which predefines the localization of the dipole sources, and the inverse solutions [34] [35]. In the current study an average head model from the reconstruction of 152 normal MRI scans (MNI template http://www.loni.ucla.edu/ICBM/) has been used, while the four different compartments of the head model (scalp, outer and inner skull, cortex) were extracted using the Boundary Element Method (BEM). BEM is implemented in the Brainstorm toolbox which is freely available in http://www.neuroimage.usc.edu/brainstorm. BEM computes the aforementioned compartments by closed triangle meshes with limited number of nodes (in our case we have used 258 nodes). Regarding the regularized solution of the linear inverse problem, we have used the column-norm normalization, resulting to a transition kernel from our 28 scalp signals to 258 cortical signals (Figure 2).

### Graph Analysis

**Figure 3:**
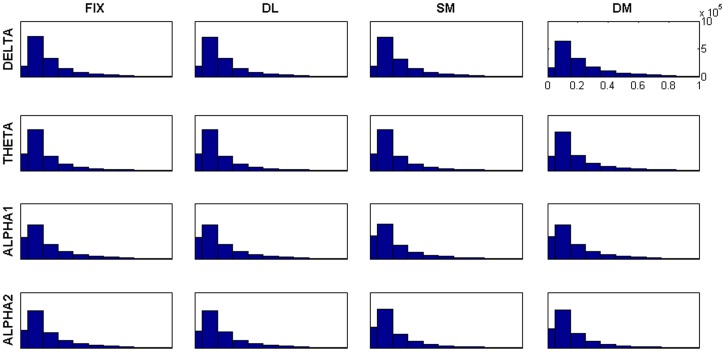
Distribution of the edges’ weights. In this figure, the distributions of the AM weights are illustrated for all tasks and for each frequency band. As it can be observed, all distributions are identical.

A weighted graph is a mathematical representation of a set of elements (vertices) that may be linked through connections of variable weights (edges). In our study, the vertices corresponded to estimated cortical dipoles. The weight of each edge is given by the Magnitude Square Coherence (MSC) value within each pair of vertices (see Appendix S1). At the group level estimation of graph parameters was conducted on a set of 672 (24 subjects×6 bands×4 tasks) Adjacency Matrices (AM). The element of the AM denotes the MSC value across the *i* ^th^ and the *j* ^th^ dipolar sources. Each matrix consisted of all 258×258 elements (estimated cortical dipoles) without applying any thresholding procedure. The distributions of the edges’ weights observed in these AMs are identical (see Figure 3 and Figure S1).

In order to investigate how each cognitive task modulates the functional connectivity of the cerebral cortex we have adopted six parameters which can describe some properties of a weighted graph. These parameters are node strength (NS), the global (Eglob) and the local (Eloc) efficiencies and the small-worldness (SWN). We have also calculated the weighted clustering coefficient (CC) and the shortest path length (L) (Table 1), but they are only mentioned in the Table 1 and they are not further discussed because their findings as well as their interpretation were similar to the Eglob and Eloc. As described in more detail in Appendix S2, Global efficiency (Eglob) is the average of the inverse of the length of the shortest path between two vertices, Local efficiency (Eloc) reflects the degree to which a graph is organized into local subgraphs, and Node strength (NS) summarizes the connection weights for a particular vertex. It should be noted that NS is a measure of regional engagement, as it flashes out the nodes with strong and dense connection with the resting nodes.

**Table 1:** Statistical results for the clustering coefficient (CC) and the characteristic path length (L).

									p-Values				
		mean			sd				FIX	DL	SM		DM
**Delta Band**													
**CC**	**FIX**	0,157		0,027				–		0,38	0,78	**0,006**	
	**DL**	0,154		0,029						–	0,76	**0,005**	
	**SM**	0,155		0,034							–	**0,012**	
	**DM**	0,182		0,044								**–**	
**L**	**FIX**	0,052		0,009				–		**0,02**	0,11	0,83	
	**DL**	0,048		0,007						–	0,55	**0,021**	
	**SM**	0,049		0,006							–	**0,06**	
	**DM**	0,051		0,007								**–**	
**Theta Band**													
**CC**	**FIX**	0,132	0,022					–		0,87	0,41	**0,01**	
	**DL**	0,131	0,024							–	0,64	**0,002**	
	**SM**	0,130	0,020								–	**0,001**	
	**DM**	0,147	0,027									–	
**L**	**FIX**	0,037	0,004					–		0,54	0,93	**0,04**	
	**DL**	0,038	0,006							–	0,61	0,25	
	**SM**	0,037	0,005								–	**0,04**	
	**DM**	0,040	0,005									–	
**Alpha1 Band**													
**CC**	**FIX**	0,123	0,022				–			0,10	0,52	0,22	
	**DL**	0,130	0,033							–	0,07	0,76	
	**SM**	0,120	0,021								–	**0,031**	
	**DM**	0,128	0,022									–	
**L**	**FIX**	0,016	0,002				–			**0,001**	0,87	0,63	
	**DL**	0,019	0,003							–	**0,001**	**0,03**	
	**SM**	0,017	0,002								–	0,68	
	**DM**	0,017	0,002									–	
**Alpha2 Band**													
**CC**	**FIX**	0,134	0,029			–				0,26	**0,015**	0,16	
	**DL**	0,139	0,031							–	**0,0004**	**0,047**	
	**SM**	0,121	0,022								–	0,06	
	**DM**	0,127	0,018									–	
**L**	**FIX**	0,03	0,006			–				0,52	**0,024**	0,14	
	**DL**	0,031	0,005							–	**0,003**	**0,06**	
	**SM**	0,027	0,004								–	0,21	
	**DM**	0,028	0,003									–	

These results are not further discussed because they don’t provided an added value, from a neuroscientific point of view at least, while they are almost identical with the results reported for global and local efficiencies.

Finally, high values on the SWN index [30], [36] reflect high clustering coefficient and short path length values which are characteristic of many real networks displaying optimum organization. The computation of graph parameters were performed using the Brain Connectivity Toolbox (BCT) [37] working on MATLAB 7.10 (The Mathworks Inc).

### Statistical Analysis

In order to check whether our variables have Gaussian distribution, Shapiro-Wilk and Kolmogorov Smirnov tests were employed. Initially, overall graph organization was quantified through the aforementioned three graph parameters, Eglob, Eloc, SWN. Both tests suggested that the distribution of individual values deviated from the normal distribution (p<.01 in all cases). Accordingly, pairwise task differences on each parameter were assessed through the asymptotic 2-tailed Mann & Witney U test. The U tests were used for the statistical comparison of the alternations in all the graph parameters mentioned above, except the NS because this measure was computed for each node separately. Thus, each task was compared in the node level using also U test, while the False Discovery Rate (FDR) method was adopted in order to control for Type I error while maintaining the nominal alpha level to.05 [38].

## Results

Table 2 shows the mean and SD values for the three graph parameters Eloc, Eglob, and SWN for each frequency band and for all tasks. FDR-corrected p values, for Mann & Witney U tests assessing pair-wise task differences on each parameter, are also shown. In delta band, the Eloc index is linearly dependent to the task’s difficulty, showing its lowest value for the control task and its highest value for the DM task. Eglob shows the same pattern with increasing values from the control situation to the difficult multiplication. In all tasks, the pattern has the characteristics of the SWN organization differing during the difficult mathematics compared to the control situation and the rest tasks. The values of theta band show also a similar pattern. Alpha 1 band shows only differences in SWN values between the DL and the SM tasks. Alpha 2 band shows the same SWN difference, alongside with a statistical significant difference on the DL and DM tasks. Beta and gamma bands show no differences in SWN and Eloc, Eglob. The weigthed clustering coefficient (CC) and the shortest path length (L) (Table 1) have almost identical findings with Eloc and Eglob respectively. Additionally they show a difference between retrieval and difficult multiplication for alpha1 band; they also discriminate the control situation from retrieval in alpha 2 band and the control situation from dot looking (the CC).

**Table 2:** Statistical results for Delta, Theta, Alpha1 and Alpha2 Brainwaves.

						p-Values					
				mean	sd	FIX			DL	SM	DM
**Delta Band**											
**SWN**	**FIX**			0,826	0,021	–			0,884	0,676	**0,012**
	**DL**			0,826	0,022				–	0,814	**0,004**
	**SM**			0,825	0,023					–	**0,004**
	**DM**			0,840	0,023						**–**
**Eglob**	**FIX**			0,250	0,024	–			0,286	0,215	**0,0009**
	**DL**			0,254	0,029				–	0,586	**0,005**
	**SM**			0,256	0,034					–	**0,017**
	**DM**			0,281	0,042						**–**
**Eloc**	**FIX**			0,175	0,024	–			0,781	0,844	**0,006**
	**DL**			0,178	0,029				–	0,958	**0,012**
	**SM**			0,180	0,034					–	**0,018**
	**DM**			0,206	0,043						–
**Theta Band**											
**SWN**	**FIX**			0,795	0,015	–			0,361	0,978	**0,004**
	**DL**			0,799	0,016				–	0,418	**0,040**
	**SM**			0,795	0,014					–	**0,001**
	**DM**			0,807	0,015						–
**Eglob**	**FIX**			0,241	0,028	–			0,988	0,787	**0,007**
	**DL**			0,241	0,026				–	0,825	**0,004**
	**SM**			0,241	0,024					–	**0,012**
	**DM**			0,256	0,032						–
**Eloc**	**FIX**			0,157	0,025	–			0,584	0,860	**0,023**
	**DL**			0,157	0,024				–	0,687	**0,042**
	**SM**			0,156	0,021					–	**0,021**
	**DM**			0,172	0,028						–
**Alpha1 Band**											
**SWN**	**FIX**		0,763		0,032	–		0,677		0,078	0,560
	**DL**		0,765		0,023			–		**0,01**	0,292
	**SM**		0,752		0,021					–	0,499
	**DM**		0,759		0,021						–
**Eglob**	**FIX**		0,242		0,028	–		0,857		0,115	0,965
	**DL**		0,242		0,026			–		0,173	0,913
	**SM**		0,242		0,024					–	**0,05**
	**DM**		0,256		0,032						–
**Eloc**	**FIX**		0,159		0,038	–		0,860		0,369	0,672
	**DL**		0,158		0,033			–		0,288	0,733
	**SM**		0,148		0,022					–	0,197
	**DM**		0,157		0,023						–
**Alpha2 Band**											
**SWN**	**FIX**	0,787			0,030	–	0,191			0,134	0,181
	**DL**	0,794			0,024		–			**0,009**	**0,020**
	**SM**	0,777			0,025					–	0,529
	**DM**	0,779			0,019						–
**Eglob**	**FIX**	0,247			0,032	–	0,231			**0,018**	0,309
	**DL**	0,251			0,031		–			**0,0006**	0,083
	**SM**	0,236			0,024					–	**0,029**
	**DM**	0,243			0,021						–
**Eloc**	**FIX**	0,161			0,033	–	0,599			0,059	0,780
	**DL**	0,165			0,031		–			**0,004**	0,227
	**SM**	0,147			0,022					–	0,105
	**DM**	0,154			0,018						–

As it was expected, the most prominent differences are observed in the comparison of the DM with the rest tasks. Considering the alternation of the graph parameters, it seems that the graphs’ organization of the aforementioned brainwaves is modulated by the engagement of the subitizing and retrieval systems.

**Figure 4:**
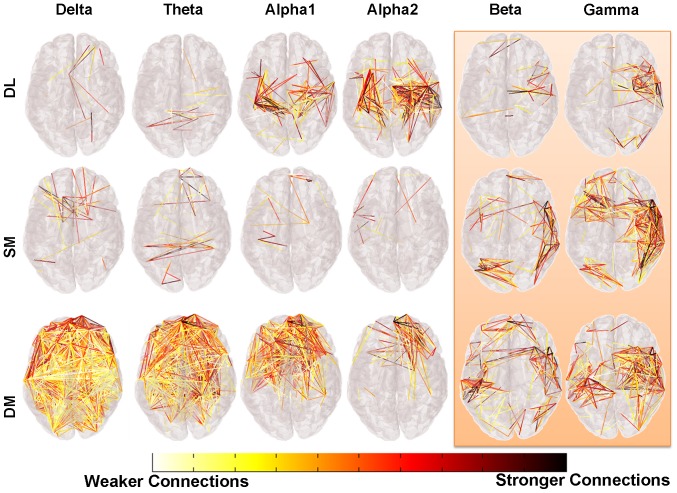
Connection differences. The color scale represents the magnitude of the difference in weighted graphs between the three examined tasks compared to the control one (FIX). The adjacency matrix of the FIX task was subtracted from the adjacency matrix of the each task and for each frequency band revealing connections that were stronger for the latter tasks. Because the herein graphs are complete we have used some thresholds (.10 for Delta, Theta, Alpha1 and Alpha2 bands and.05 for Beta and Gamma bands) only for the illustration of the engagement of the subitizing and retrieval subsystems.

**Figure 5:**
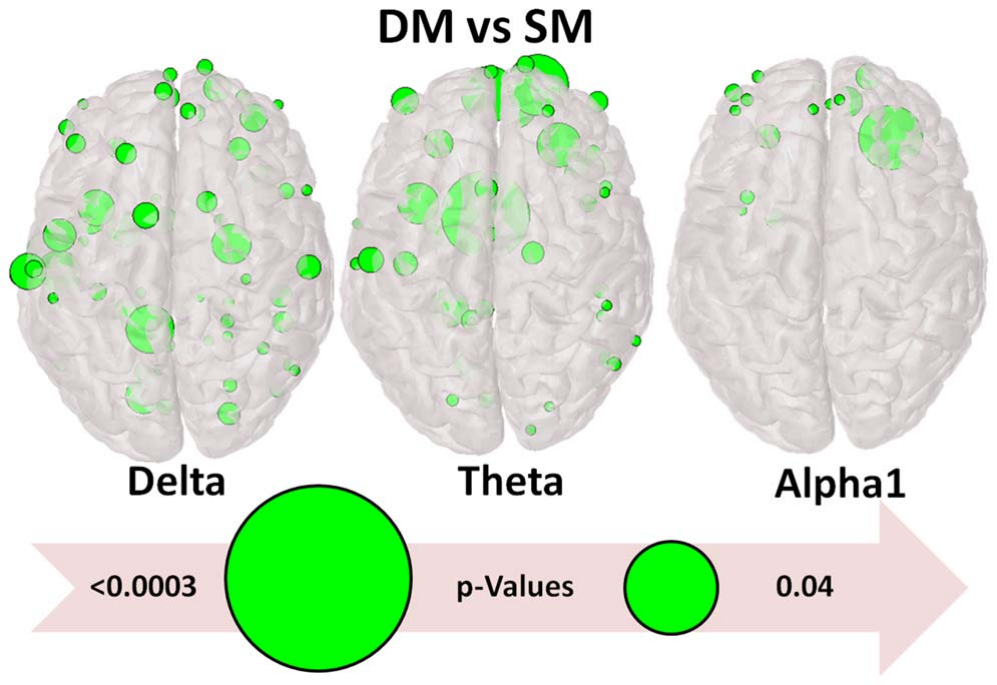
Statistical Significant Differences of NS. Nodes found to be significantly stronger for the DM than the SM task at three frequency bands. The size of green nodes is inversely proportional to the significant (<.05) p-values: the larger the node the more significant the effect is.

**Figure 6:**
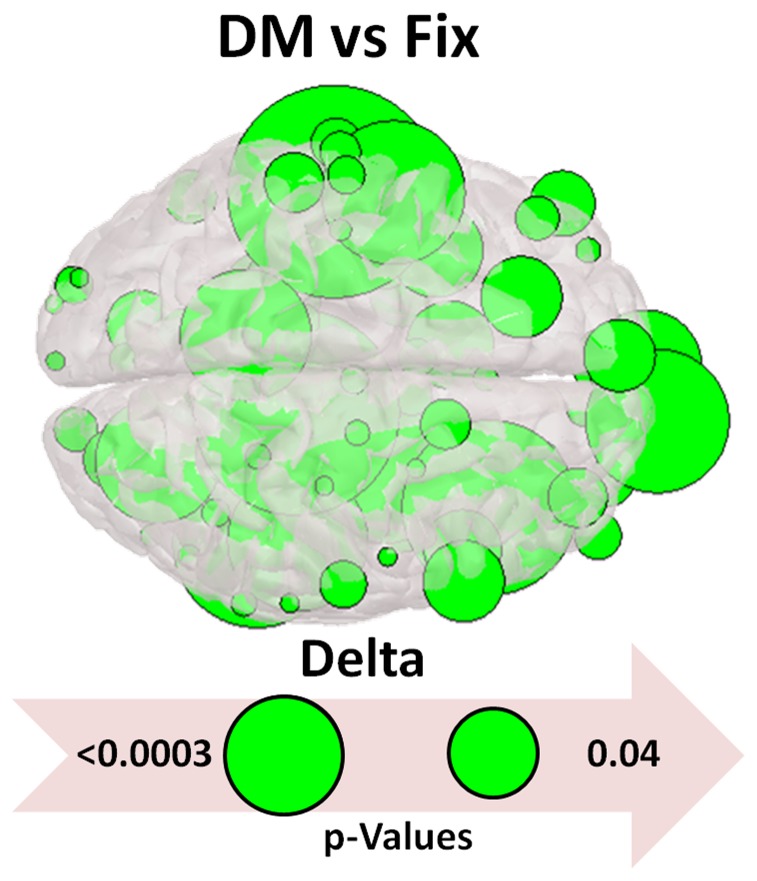
Node Strength among the DM and FIX tasks. This figure was extracted like the Figure 5, with the only difference that here we compare the DM with the FIX tasks in the delta band. The size of green nodes is inversely proportional to the significant (<.05) p-values: the larger the node the more significant the effect is.

The most striking differences were found during DM. Fig 4 illustrates the strength of connections in the different bands in weighted graphs. These are represented in a low threshold to visualize weaker ties and in high threshold to include stronger ties. For the high frequency bands we used low threshold. Delta and theta bands show a similar pattern with prominent (weighted) edges more frontal and fronto-parietal. A similar pattern shows alpha 1 and alpha 2 bands, but with fewer prominent edges towards the occipital regions. The higher frequency bands which are of cortical origin and are activated locally show the most prominent edges in temporal, temporoparietal and towards occipital regions. Moreover, Figs 5 and 6 show higher values of the node strength (an index of node connections) during the difficult multiplication task, more frontal and more on the left hemisphere. Finally we would like to report that the results didn’t reveal any statistical significant difference as it can be observed in Table 3.

**Table 3:** Statistical results for Beta and Gamma Bands.

				p-Values				
		mean	sd	FIX	DL		SM	DM
**Beta Band**								
**SWN**	**FIX**	0,826728	0,012205	–	0,847		0,867	0,399
	**DL**	0,826184	0,012957		–		0,995	0,616
	**SM**	0,826204	0,014302				–	0,552
	**DM**	0,824631	0,013091					–
**Eglob**	**FIX**	0,217919	0,027464	–	0,177		0,193	0,677
	**DL**	0,212669	0,020283		–		0,782	0,120
	**SM**	0,213138	0,020402				–	0,122
	**DM**	0,217006	0,023572					–
**Eloc**	**FIX**	0,14579	0,021042	–	0,718		0,657	1
	**DL**	0,142066	0,015834		–		0,893	0,516
	**SM**	0,142368	0,017031				–	0,627
	**DM**	0,14506	0,017661					–
**Gamma Band**								
**SWN**	**FIX**	0,81255	0,014384	–		0,104	0,245	0,311
	**DL**	0,816167	0,012885			–	0,461	0,819
	**SM**	0,81434	0,011113				–	0,710
	**DM**	0,815431	0,012426					–
**Eglob**	**FIX**	0,231598	0,033379	–		0,070	0,206	0,779
	**DL**	0,222402	0,018434			–	0,423	0,066
	**SM**	0,224617	0,021325				–	0,150
	**DM**	0,230667	0,027769					–
**Eloc**	**FIX**	0,154207	0,027926	–		0,502	0,556	0,764
	**DL**	0,147176	0,015078			–	0,975	0,269
	**SM**	0,148614	0,017083				–	0,369743
	**DM**	0,154713	0,024452					–

It seems that Beta and Gamma brainwaves are not involved in the organization of the subitizing and retrieval systems.

## Discussion

In this study network analysis was used to compare the two core systems of mathematical function with a difficult calculation estimating the effects on the different EEG bands. It was observed that at rest and during the mathematical tasks, the involved brain networks showed the optimum (SWN) organization for all frequency bands and tasks. The graph parameters for delta and theta bands, and in a lesser degree for the alpha 2 band, seem to have some statistically significant differences during mathematical cognitive tasks with increasing difficulty.

Delta band increases in pathological brain disturbances and during sleep. Oscillations in delta range are believed to be generated by neocortical and thalamocortical networks [39]. This band is important for large scale cortical integration [40]. During cognitive processes, changes on delta band are produced and their degree depends on the complexity of tasks [41]. Decreases of delta activity have been recorded in conditions that require attention to the external environment, whereas increases have been manifested during cognitive processes requiring attention to internal processing [42], [43]. In mathematical calculations, increases of delta activity have also been reported [41], [42], [44]–[47]. The increase of the delta band is believed to be related with inhibitory mechanisms [42] and especially with inhibition of the default mode network. This inhibition has also been observed in other studies of mental calculation using fMRI or bioelectrical signals [44], [48], [49]. In the present study, interesting findings are related to network organizations of delta band during the mathematical tasks. The increasing values of Eloc and Eglob from subitizing to the retrieval of simple multiplication and difficult multiplication show increasing nodal organization and efficient remote connectness. During the difficult multiplication, the higher cliquish organization (higher Eloc) could be indicative of higher and more specific demands; moreover, the integration (Eglob) is more effective. This is an interesting finding that depends not only on the role of delta band for large scale cortical integration. The network reactivity of delta band shows more prominent (weighted) connections in frontal/frontoparietal regions in higher thresholds (Fig. 4). Additionally, there is a hemispheric asymmetry with higher activation of the left hemisphere during the difficult task (see Figures 5, 6, S2, S3, S4) which comes into accordance with previous studies [50].

Theta band increases during sleep stage I, but also during many cognitive tasks and especially during numerous working memory processes [51], [52]. Additionally, theta band synchronizations are visible during remote dynamic information transfers between distant cortical regions; theta band oscillations during cognitive tasks are visible mainly at the frontal midline regions [43], [51], but they can be also present on parietal and temporal sites [53]. Their power depends on the cognitive load. Theta band coherence between frontal and parietal sites has also been described in recent literature [40], [51]. In mental arithmetic tasks, enhancement of frontal theta activity [54], [55] or synchronization in a large number of electrode pairs have also been reported [53]. Our findings are indicative of high nodal and remote organization during the difficult multiplication task. The Eloc value was highest during the difficult multiplication task and Eglob during the dot looking task. The mapping showed more prominent connections in frontoparietal regions. Node strength showed more prominent nodes in frontal regions of the left hemisphere. These are in accordance with previous studies [23], [50], [56]. Thus, theta band network parameters can describe mathematical tasks and add information in accordance with previous studies [44], [46].

Alpha 1 band oscillations are of corticothalamic origin and are the most well-studied EEG oscillations. Increased cortical activity is accompanied with a decrease of alpha 1 activity. Increases of alpha2 activity are described in combination with increases of theta band. The mapping of the (weighted) interconnections in this piece of work, were stronger in frontal and frontoparietal regions and the node strength was higher frontally on the right side for the alpha 1 band. Eloc and Eglob showed only a few differences in alpha2 during difficult multiplications (Eglob) and retrieval (Eloc). The frontoparietal finding is the result of working memory load [51], [52].

High frequency bands that are of cortical origin and are activated locally, showed connections with high temporal/temporoparietal strengths during the difficult mathematical tasks.

Altogether, the network analysis of the different EEG bands of core mathematical tasks and comparisons with difficult mathematical calculations, showed differences in network organization especially in low frequency bands. Mental arithmetic is a complex mental task differing from the core systems (subitizing, retrieval). The core system tasks are simple functions especially subitizing. This is an evolutionally ancient system; it is an “automatic” and efficient function which is evident in the minimum reactivity shown by the graph parameters in comparison to the other tasks. The retrieval is an automatic simple process and the brain function is more efficient differing from the difficult multiplication task. The difficult multiplication task is a complex mental act requiring the integration of multiple cognitive processes. It differs from the other tasks as seen from the delta and theta band reactivity. Delta band reactivity could be the result of higher Default Mode Network (DMN) reactivity, while theta band reactivity could be seen as the result of working memory activations mainly. Additional interesting findings are prominent in fronto-parietal connections for delta, theta, alpha 1 and alpha2 bands with temporal-temporoparietal enhancements for beta and gamma bands. The node strength shows prominent frontal activity and on the left hemisphere for the lower frequency bands.

As a conclusion, the network analysis presented herein, seems to be a quite useful tool upon describing cognitive processes such as mental arithmetic tasks. It is, therefore, expected that it could be used to study disturbances in arithmetic ability, such as dyscalculia. Thus, a good idea for a future research is the study of the cortical networks obtained by adults suffering from dyscalculia during mental arithmetics. Their dysfunction of subitizing and retrieval systems are expected to alter the formed networks, while it is assumed that this departure from the results presented herein will provide further evidence for the functionality of the aforementioned systems [57].

.

## Supporting Information

Figure S1 Boxplots of the distribution of the edges’ weights.
(TIF)

Figure S2 The hemispheric differences for Delta and Theta Bands.
(TIF)

Figure S3 The hemispheric differences for Alpha1 and Alpha2 Bands.
(TIF)

Figure S4 Node Strength among all tasks. This figure was extracted like the Figures 5 and 6 in the manuscript. The size of green nodes is inversely proportional to the significant (<.05) p-values: the larger the node the more significant the effect is.
(TIF)

Appendix S1 (DOCX)

Appendix S2 (DOCX)
