# Supplementary figures and images for: A Graph Theoretical Approach to Study the Organization of the Cortical Networks during Different Mathematical Tasks
Source: PLoS One. 2013 Aug 19;8(8):e71800. doi: 10.1371/journal.pone.0071800 (PMC3747176; doi:10.1371/journal.pone.0071800)

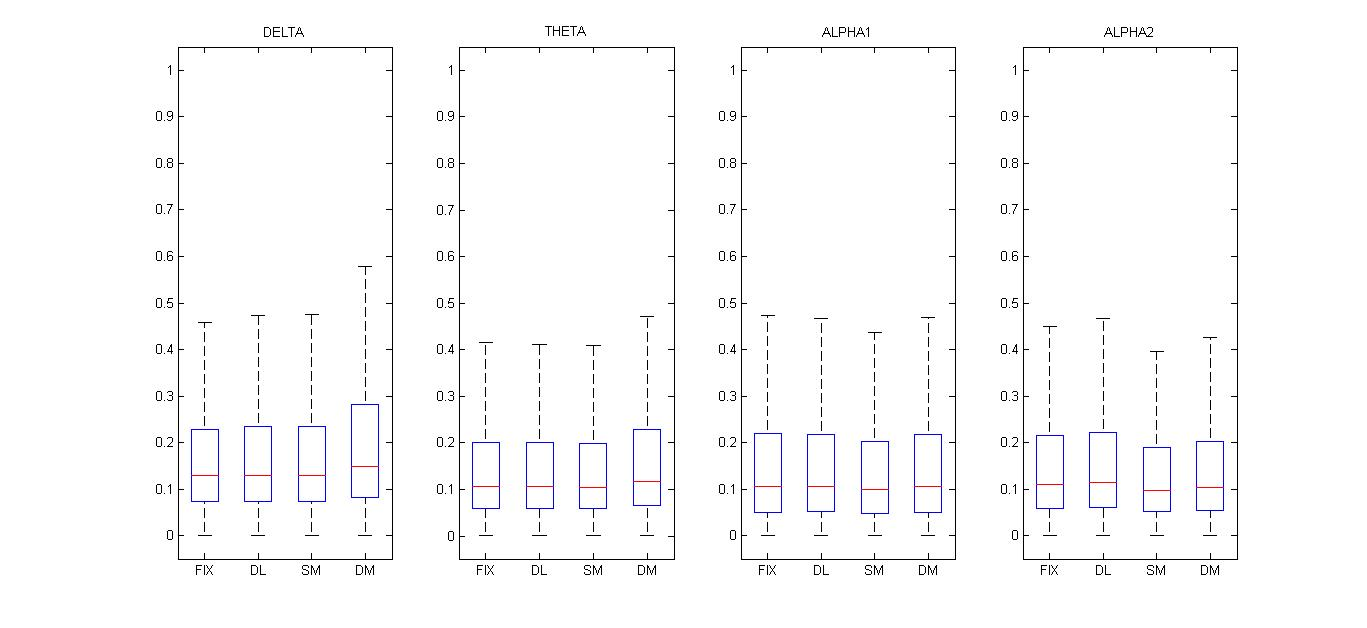

Supplement: Figure S1 — Boxplots of the distribution of the edges’ weights. (TIF) [file pone.0071800.s001.tif]

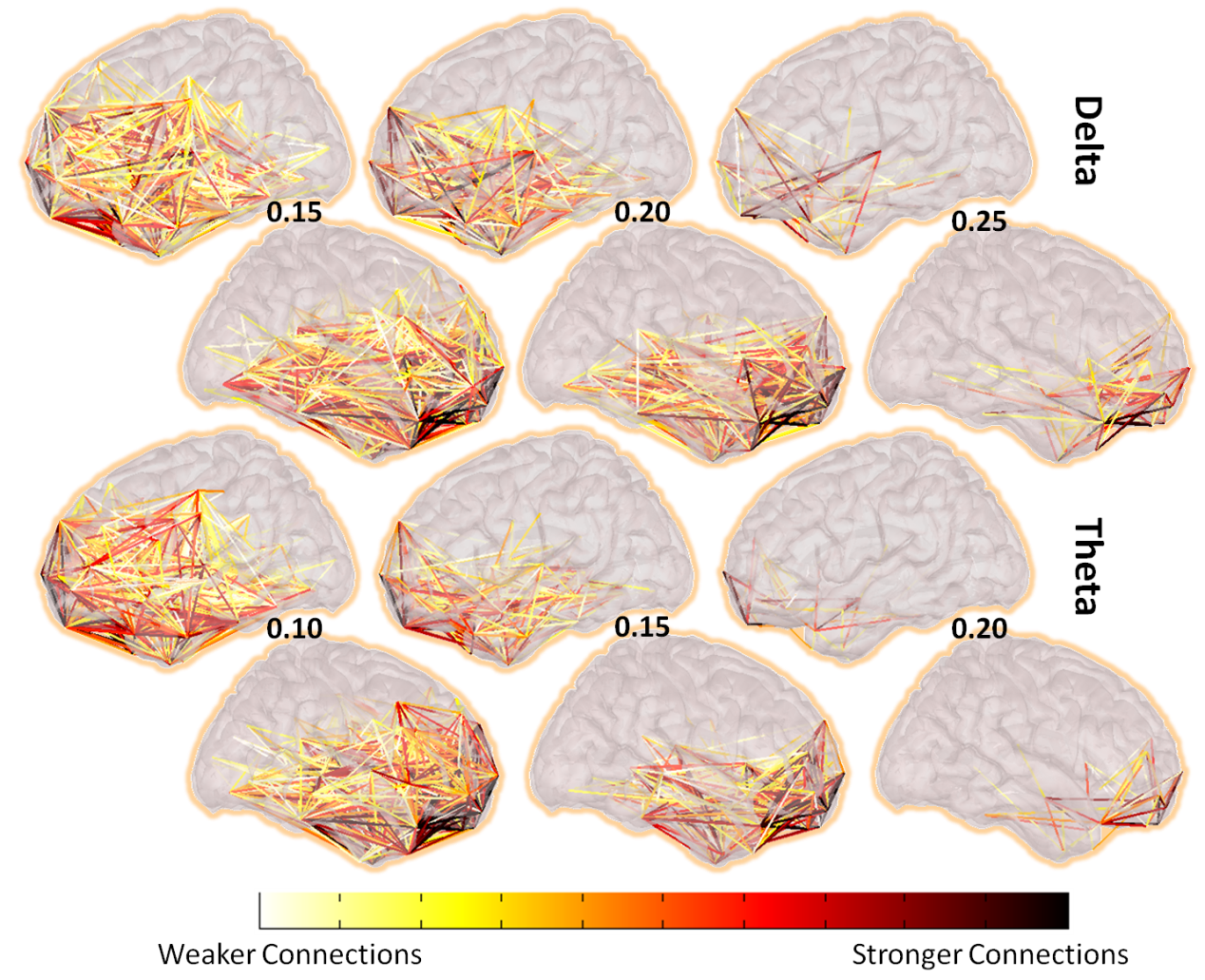

Supplement: Figure S2 — The hemispheric differences for Delta and Theta Bands. (TIF) [file pone.0071800.s002.tif]

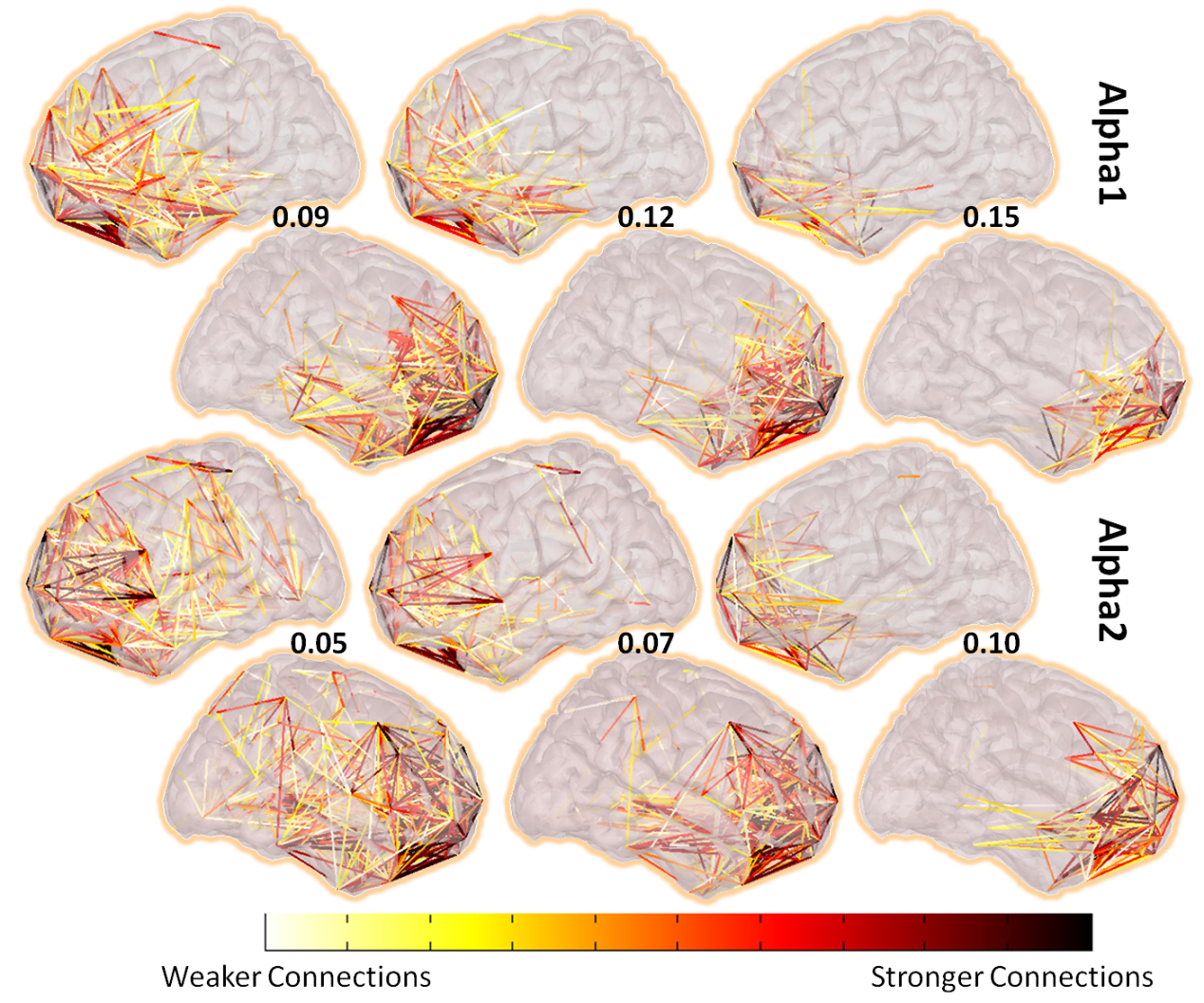

Supplement: Figure S3 — The hemispheric differences for Alpha1 and Alpha2 Bands. (TIF) [file pone.0071800.s003.tif]

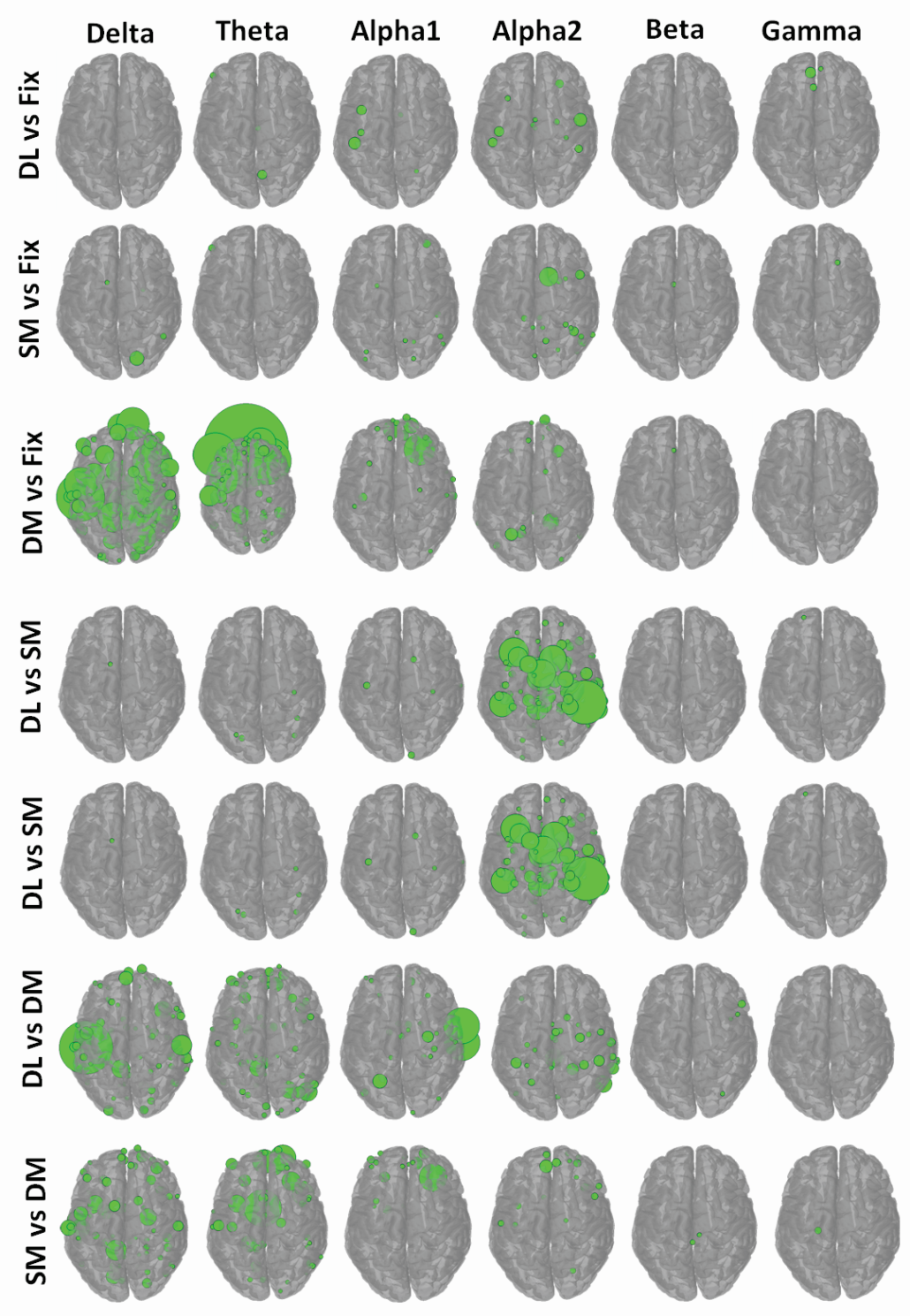

Supplement: Figure S4 — Node Strength among all tasks. This figure was extracted like the Figures 5 and 6 in the manuscript. The size of green nodes is inversely proportional to the significant (<.05) p-values: the larger the node the more significant the effect is. (TIF) [file pone.0071800.s004.tif]
