# Supplementary material for: A Graph Theoretical Approach to Study the Organization of the Cortical Networks during Different Mathematical Tasks
Source: PLoS One. 2013 Aug 19;8(8):e71800. doi: 10.1371/journal.pone.0071800 (PMC3747176; doi:10.1371/journal.pone.0071800)
Supplement: Appendix S1 — (DOCX) [file pone.0071800.s005.docx]

**Appendix S1 (Functional Connectivity)**

Functional connectivity of the cortical sources was estimated using the Magnitude Square Coherence (MSC) function of MATLAB v. 7.10 (The MathWorks Inc.), based on recent evidence that it is more suitable to model cerebral networks compared to other connectivity measures [1]. The MSC (1) in a particular frequency ($f$) is defined as the square of the cross Power Spectrum Density (PSD) of signals $x$ and $y$divided by the product of the PSDs of $x$ and $y$ respectively.

$MSC_{xy}(f)=\frac{\left|P_{xy}(f)\right|^{2}}{P_{xx}(f)P_{yy}(f)}$ (1)

The PSD was estimated using the Welch method [2]. The signals were divided into segments containing 400 samples each, and PSD was then computed using the formula:

$P(f)=\frac{1}{f_{s}L_{s}U}\int_{-f_{s}/2}^{f_{s}/2} D_{xx}(\rho)\left|W\left(f-\rho\right)\right|^{2}d\rho$ (2)

where $D_{xx}$ is the Discrete Fourier Transform of the signal’s correlation sequence $R_{xx}$, $f_{s}$ is the digitization rate (here 500 Hz), $L_{s}$ is the segment length and $U$(3) is a normalization constant ensuring that the PSD is asymptotically unbiased.

1. Lithari C, Klados MA, Papadelis C, Pappas C, Albani M, et al. (2011) How does the metric choice affect brain functional connectivity networks?, Biomedical Signal Processing and Control, Available online 14 October, ISSN 1746-8094, 10.1016/j.bspc.2011.05.004.
2. Welch, P.D. (1967) "The Use of Fast Fourier Transform for the Estimation of Power Spectra: A Method Based on Time Averaging Over Short, Modified Periodograms", IEEE Transactions on Audio Electroacoustics, AU-15, 70–73.
