# Supplementary material for: A Graph Theoretical Approach to Study the Organization of the Cortical Networks during Different Mathematical Tasks
Source: PLoS One. 2013 Aug 19;8(8):e71800. doi: 10.1371/journal.pone.0071800 (PMC3747176; doi:10.1371/journal.pone.0071800)
Supplement: Appendix S2 — (DOCX) [file pone.0071800.s006.docx]

**Appendix S2 (Graph Parameters)**

## Global Efficiency

Latora and Marchiori [1] defined the efficiency of the path between two vertices as the inverse of their distance. In the absence of a path connecting two vertices their distance is infinite and the corresponding efficiency is 0. Global efficiency (Eglob) is computed by the following formula:

$$E_{g}=\frac{1}{n}\sum_{i\in N} \frac{\sum_{j\in N,j\neq i} \frac{1}{d_{ij}}}{n-1}$$

In our fully connected and weighted graphs the distance ($d(i,j)$) is equal to the length of the shortest path, where the path’s length among two nodes is the inverse of their edge’s weight.

## Local Efficiency

Local efficiency (Eloc) ($E_{i}$) of a graph is the global efficiency of the subgraph $S_{i}$. This subgraph is formed by removing the $i$^th^ node, and taking the remaining nodes which were connected to the removed one. Local efficiency reflects the tendency of a graph to form clusters **Error! Reference source not found.**. In this sense, it is understandable that the local efficiency of a fully connected graph equals 1, while the local efficiency of an empty graph is 0. In weighted graphs local efficiency can be computed by the following formula:

In our analysis we have computed the Eloc for every node and then we took their mean value in order to extract a characteristic value for the whole graph.

## Small Worldness

In science, a small-world (SWN) network is a graph in which most nodes are not neighbors of one another, but most nodes can be reached from every other by a small number of steps. The [2] have defined the small-worldness of a network as:

$$S=\frac{C/C_{rand}}{L/L_{rand}}$$

where care the cluster coefficient and the short path length respectively, while the $C_{rand},L_{rand}$indices define the mean values of $C$and $L$respectively, extracted by fifty surrogate random networks [3]. Each random network was produced by a random rewiring of the observed network [4] . Regarding the computation of the weighted clustering coefficient, there are at least four different definitions [5]. For the purposes of the current analysis we have used the one proposed by Onnela et al. [6] because it takes into account the weights of all edges in a triangle, as well as it is invariant to the weight’s permutation in a single triangle.

## Node Strength

An important measure of network structure that summarizes connection weights is node strength, an extension of node degree. Node strength is defined as the sum of weights of all the vertices connected to a certain node according to the following formula:

$$ns_{i}=\sum_{j=1}^{N} a_{ij}w_{ij}$$

where $ns_{i}$is the node strength of the $i$^th^ node, a_ij_ equals 1 if the $i$^th^ and $j$^th^ nodes are connected and 0 otherwise, and $w_{ij}$ is the weight of the connection among the $i$^th^ and $j$^th^ nodes.

1. Latora V, Marchiori, M. (2001) Efficient Behavior of Small-World Networks. Phys. Rev. Lett. 87, 198701
2. Humphries MD, Gurney K (2008) Network ‘Small-World-Ness’: A Quantitative Method for Determining Canonical Network Equivalence. PLoS ONE 3(4): e0002051. doi:10.1371/journal.pone.0002051
3. Vasso Tsirka, Panagiotis G. Simos, Antonios Vakis, Kassiani Kanatsouli, Michael Vourkas, et al., (2011) Mild traumatic brain injury: Graph-model characterization of brain networks for episodic memory, International Journal of Psychophysiology, Volume 79, Issue 2, Pages 89-96, ISSN 0167-8760, 10.1016/j.ijpsycho.2010.09.006.
4. Zalesky, A., Fornito, A., & Bullmore, E. (2012). On the use of correlation as a measure of network connectivity. NeuroImage, 60(4), 2096-2106. Elsevier Inc. doi:10.1016/j.neuroimage.2012.02.001
5. J Saramäki, M Kivelä, JP Onnela, K Kaski, J Kertesz, (2007) Generalizations of the clustering coefficient to weighted complex networks, Phys. Rev. E 75, 027105.
6. J.-P. Onnela, J. Saramäki, J. Kertész, and K. Kaski, (2005) Intensity and coherence of motifs in weighted complex networks, Physical Review E 71, 065103.
